# Deep Learning in Label-free Cell Classification

**DOI:** 10.1038/srep21471

**Published:** 2016-03-15

**Authors:** Claire Lifan Chen, Ata Mahjoubfar, Li-Chia Tai, Ian K. Blaby, Allen Huang, Kayvan Reza Niazi, Bahram Jalali

**Affiliations:** 1 Department of Electrical Engineering, University of California, Los Angeles, California 90095, USA; 2 California NanoSystems Institute, Los Angeles, California 90095, USA; 3 Department of Chemistry and Biochemistry, University of California, Los Angeles, California 90095; 4 NantWorks, LLC, Culver City, California 90232, USA; 5 Department of Bioengineering, University of California, Los Angeles, California 90095, USA; 6 Department of Surgery, David Geffen School of Medicine, University of California, Los Angeles, California 90095, USA

## Abstract

Label-free cell analysis is essential to personalized genomics, cancer diagnostics, and drug development as it avoids adverse effects of staining reagents on cellular viability and cell signaling. However, currently available label-free cell assays mostly rely only on a single feature and lack sufficient differentiation. Also, the sample size analyzed by these assays is limited due to their low throughput. Here, we integrate feature extraction and deep learning with high-throughput quantitative imaging enabled by photonic time stretch, achieving record high accuracy in label-free cell classification. Our system captures quantitative optical phase and intensity images and extracts multiple biophysical features of individual cells. These biophysical measurements form a hyperdimensional feature space in which supervised learning is performed for cell classification. We compare various learning algorithms including artificial neural network, support vector machine, logistic regression, and a novel deep learning pipeline, which adopts global optimization of receiver operating characteristics. As a validation of the enhanced sensitivity and specificity of our system, we show classification of white blood T-cells against colon cancer cells, as well as lipid accumulating algal strains for biofuel production. This system opens up a new path to data-driven phenotypic diagnosis and better understanding of the heterogeneous gene expressions in cells.

Deep learning extracts patterns and knowledge from rich multidimenstional datasets. While it is extensively used for image recognition and speech processing, its application to label-free classification of cells has not been exploited. Flow cytometry is a powerful tool for large-scale cell analysis due to its ability to measure anisotropic elastic light scattering of millions of individual cells as well as emission of fluorescent labels conjugated to cells^1^,^2^. However, each cell is represented with single values per detection channels (forward scatter, side scatter, and emission bands) and often requires labeling with specific biomarkers for acceptable classification accuracy^1^,^3^. Imaging flow cytometry^4^,^5^ on the other hand captures images of cells, revealing significantly more information about the cells. For example, it can distinguish clusters and debris that would otherwise result in false positive identification in a conventional flow cytometer based on light scattering^6^.

In addition to classification accuracy, the throughput is another critical specification of a flow cytometer. Indeed high throughput, typically 100,000 cells per second, is needed to screen a large enough cell population to find rare abnormal cells that are indicative of early stage diseases. However there is a fundamental trade-off between throughput and accuracy in any measurement system^7^,^8^. For example, imaging flow cytometers face a throughput limit imposed by the speed of the CCD or the CMOS cameras, a number that is approximately 2000 cells/s for present systems^9^. Higher flow rates lead to blurred cell images due to the finite camera shutter speed. Many applications of flow analyzers such as cancer diagnostics, drug discovery, biofuel development, and emulsion characterization require classification of large sample sizes with a high-degree of statistical accuracy^10^. This has fueled research into alternative optical diagnostic techniques for characterization of cells and particles in flow.

Recently, our group has developed a label-free imaging flow-cytometry technique based on coherent optical implementation of the photonic time stretch concept^11^. This instrument overcomes the trade-off between sensitivity and speed by using Amplified Time-stretch Dispersive Fourier Transform^12^,^13^,^14^,^15^. In time stretched imaging^16^, the object’s spatial information is encoded in the spectrum of laser pulses within a pulse duration of sub-nanoseconds (Fig. 1). Each pulse representing one frame of the camera is then stretched in time so that it can be digitized in real-time by an electronic analog-to-digital converter (ADC). The ultra-fast pulse illumination freezes the motion of high-speed cells or particles in flow to achieve blur-free imaging. Detection sensitivity is challenged by the low number of photons collected during the ultra-short shutter time (optical pulse width) and the drop in the peak optical power resulting from the time stretch. These issues are solved in time stretch imaging by implementing a low noise-figure Raman amplifier within the dispersive device that performs time stretching^8^,^11^,^16^. Moreover, warped stretch transform^17^,^18^ can be used in time stretch imaging to achieve optical image compression and nonuniform spatial resolution over the field-of-view^19^. In the coherent version of the instrument, the time stretch imaging is combined with spectral interferometry to measure quantitative phase and intensity images in real-time and at high throughput^20^. Integrated with a microfluidic channel, coherent time stretch imaging system in this work measures both quantitative optical phase shift and loss of individual cells as a high-speed imaging flow cytometer, capturing 36 million images per second in flow rates as high as 10 meters per second, reaching up to 100,000 cells per second throughput.

On another note, surface markers used to label cells, such as EpCAM^21^, are unavailable in some applications; for example, melanoma or pancreatic circulating tumor cells (CTCs) as well as some cancer stem cells are EpCAM-negative and will escape EpCAM-based detection platforms^22^. Furthermore, large-population cell sorting opens the doors to downstream operations, where the negative impacts of labels on cellular behavior and viability are often unacceptable^23^. Cell labels may cause activating/inhibitory signal transduction, altering the behavior of the desired cellular subtypes, potentially leading to errors in downstream analysis, such as DNA sequencing and subpopulation regrowth. In this way, quantitative phase imaging (QPI) methods^24^,^25^,^26^,^27^ that categorize unlabeled living cells with high accuracy are needed. Coherent time stretch imaging is a method that enables quantitative phase imaging at ultrahigh throughput for non-invasive label-free screening of large number of cells.

In this work, the information of quantitative optical loss and phase images are fused into expert designed features, leading to a record label-free classification accuracy when combined with deep learning. Image mining techniques are applied, for the first time, to time stretch quantitative phase imaging to measure biophysical attributes including protein concentration, optical loss, and morphological features of single cells at an ultrahigh flow rate and in a label-free fashion. These attributes differ widely^28^,^29^,^30^,^31^ among cells and their variations reflect important information of genotypes and physiological stimuli^32^. The multiplexed biophysical features thus lead to information-rich hyper-dimensional representation of the cells for label-free classification with high statistical precision.

We further improved the accuracy, repeatability, and the balance between sensitivity and specificity of our label-free cell classification by a novel machine learning pipeline, which harnesses the advantages of multivariate supervised learning, as well as unique training by evolutionary global optimization of receiver operating characteristics (ROC). To demonstrate sensitivity, specificity, and accuracy of multi-feature label-free flow cytometry using our technique, we classified (1) *OT-II* hybridoma T-lymphocytes and *SW-480* colon cancer epithelial cells, and (2) Chlamydomonas reinhardtii algal cells (herein referred to as Chlamydomonas) based on their lipid content, which is related to the yield in biofuel production. Our preliminary results show that compared to classification by individual biophysical parameters, our label-free hyperdimensional technique improves the detection accuracy from 77.8% to 95.5%, or in other words, reduces the classification inaccuracy by about five times.

## Results

### Time Stretch Quantitative Phase Imaging

**Figure 1:**
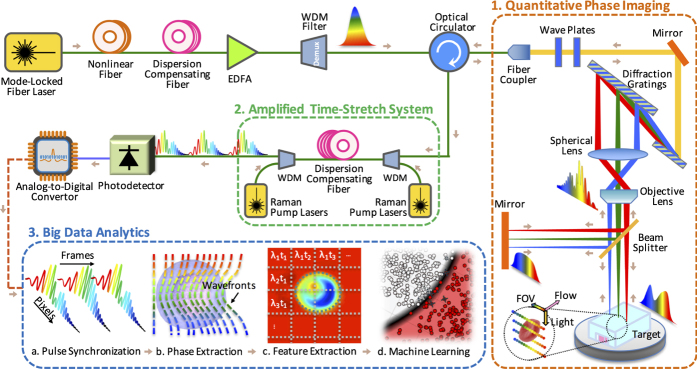
Time stretch quantitative phase imaging (TS-QPI) and analytics system; A mode-locked laser followed by a nonlinear fiber, an erbium doped fiber amplifier (EDFA), and a wavelength-division multiplexing (WDM) filter generate and shape a train of broadband optical pulses. Box 1: The pulse train is spatially dispersed into a train of rainbow flashes illuminating the target as line scans. The spatial features of the target are encoded into the spectrum of the broadband optical pulses, each representing a one-dimensional frame. The ultra-short optical pulse illumination freezes the motion of cells during high speed flow to achieve blur-free imaging with a throughput of 100,000 cells/s. The phase shift and intensity loss at each location within the field of view are embedded into the spectral interference patterns using a Michelson interferometer. Box 2: The interferogram pulses were then stretched in time so that spatial information could be mapped into time through time-stretch dispersive Fourier transform (TS-DFT), and then captured by a single pixel photodetector and an analog-to-digital converter (ADC). The loss of sensitivity at high shutter speed is compensated by stimulated Raman amplification during time stretch. Box 3: (a) Pulse synchronization; the time-domain signal carrying serially captured rainbow pulses is transformed into a series of one-dimensional spatial maps, which are used for forming line images. (b) The biomass density of a cell leads to a spatially varying optical phase shift. When a rainbow flash passes through the cells, the changes in refractive index at different locations will cause phase walk-off at interrogation wavelengths. Hilbert transformation and phase unwrapping are used to extract the spatial phase shift. (c) Decoding the phase shift in each pulse at each wavelength and remapping it into a pixel reveals the protein concentration distribution within cells. The optical loss induced by the cells, embedded in the pulse intensity variations, is obtained from the amplitude of the slowly varying envelope of the spectral interferograms. Thus, quantitative optical phase shift and intensity loss images are captured simultaneously. Both images are calibrated based on the regions where the cells are absent. Cell features describing morphology, granularity, biomass, etc are extracted from the images. (d) These biophysical features are used in a machine learning algorithm for high-accuracy label-free classification of the cells.

The application of time stretch quantitative phase imaging (TS-QPI) to imaging flow cytometry has been recently demonstrated in our group^11^. Broadband optical pulses from a mode-locked laser were firstly conditioned in fiber optics and then spatially dispersed in free-space optics with a pair of reflection diffraction gratings creating 1-D “rainbow flashes” (Fig. 1). Each of the rainbow flashes was composed of all the wavelength components distributed laterally over the field of view. These flashes illuminated the target as in traditional photography, but in addition, rainbow flashes targeted different spatial points with distinct colors of light, resulting in space-to-spectrum encoding. Rainbow pulses were then split into the two arms of a Michelson interferometer. Different wavelength components of the rainbow flash traveled parallel to each other but individually focused on the mirror in the reference arm or on the reflective substrate of a microfluidic device in the sample arm. In the sample arm, the cells in the microfluidic channel were hydrodynamically focused^33^,^34^ into the rainbow’s field of view and flowed perpendicular to the rainbow flash. Reflected pulses from the microfluidic device and the reference arm were recombined and coupled back into the fiber, optically amplified and linearly chirped through Raman-amplified time-stretch dispersive Fourier transform (TS-DFT) system. An amplified time-stretch system that utilizes a low-noise distributed Raman amplifier within dispersive fiber with a net optical gain of approximately 15 dB enables high-sensitivity detection at high speeds. An ultrafast single-pixel photodetector transformed instantaneous optical power into an electrical signal and subsequently, an analog-to-digital converter (ADC) samples and quantizes the signal. Acquired data are passed down to processing stages for big data analytics. The interference between time-shifted linearly chirped pulses create a beat (fringe) frequency, which can be adjusted via the interferometer arm length mismatch. Details of the demonstration system can be found in Methods: Time Stretch Quantitative Phase Imaging (TS-QPI) System.

The photodetected time-stretched pulses, each representing one line scan, are converted to analytic signals using Hilbert transformation^35^ and the intensity and phase components are extracted. The phase component is a fast oscillating fringe (carrier frequency), caused by the interference of the linearly chirped pulses arriving from the reference and sample arms in the Michelson interferometer. Acting as a radio-frequency (RF) carrier whose frequency is set by the user adjusted arm length mismatch, the fringe frequency is modulated when the optical path length in the sample arm is changed by the arrival of a cell. This frequency shift and the accompanying phase change are used to measure the optical path length of the cell (see Section Methods: Coherent Detection and Phase Extraction). Since the phase varies over a wide range (much larger than 2*π* radians), an unwrapping algorithm is used to obtain the continuous phase profile. The phase profile contains the phase shift induced by the cell and an increasing term with time, corresponding to the fringe (beat) frequency. By eliminating the background phase component, the cell-induced phase shift is extracted. The second component in the waveform is a lower frequency envelope corresponding to temporal shape of the optical pulse. The amplitude of this envelope provides information about optical loss caused by transparency, surface roughness, and inner organelle complexity (Section Methods: Cell Transmittance Extraction).

### Feature Extraction

**Figure 2 f2:**
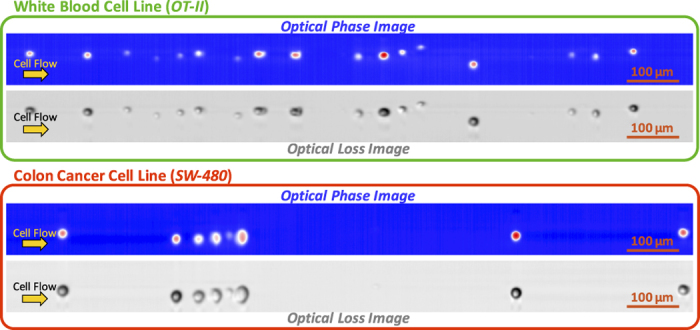
Quantitative optical phase and loss images of *OT-II* (green box) and *SW-480* (red box) cells. The optical loss images of the cells are affected by the attenuation of multiplexed wavelength components passing through the cells. The attenuation itself is governed by the absorption of the light in cells as well as the scattering from the surface of the cells and from the internal cell organelles. The optical loss image is derived from the low frequency component of the pulse interferograms. The optical phase image is extracted from the analytic form of the high frequency component of the pulse interferograms using Hilbert Transformation, followed by a phase unwrapping algorithm. Details of these derivations can be found in Section Methods. Also, supplementary Videos 1 and 2 show measurements of cell-induced optical path length difference by TS-QPI at four different points along the rainbow for *OT-II* and *SW-480*, respectively.

The decomposed components of sequential line scans form pairs of spatial maps, namely, optical phase and loss images as shown in Fig. 2 (see Section Methods: Image Reconstruction). These images are used to obtain biophysical fingerprints of the cells^8^,^36^. With domain expertise, raw images are fused and transformed into a suitable set of biophysical features, listed in Table 1, which the deep learning model further converts into learned features for improved classification.

The feature extraction operates on optical phase and loss images simultaneously, including object detection, segmentation, and feature measurement, as well as clump identification, noise suppression, etc. As an example of the expert designed features, the average refractive index, used as a measure of protein concentration^37^, is obtained by dividing the integral of the optical path length by the cell volume. Since cells in suspension relax to a spherical shape (due to surface tension)^38^,^39^, an independent measure of cell diameter can be obtained from its lateral dimension for volume estimation.

In feature extraction, one of the most important advantages of optical loss and phase fusion, is its robustness and insensitivity to axial defocusing^40^ caused by the limited depth-of-focus of the objective lens and variations of the cell alignment in microfluidic channel. Diffracted photons have little chance to be influential in phase images. This makes the size measurements in optical phase images relatively accurate and consistent, more suitable than direct size measurements in optical loss images for extraction of scattering and absorption features. Among different features, size measurement is particularly important as it is used by itself in many technologies^31^,^41^,^42^,^43^.

The large data set captured by TS-QPI provides sufficient statistical characteristics for cell analysis based on biophysical features. Since cells from even the same line or tissue exhibit variations in size, structure, and protein expression levels^44^,^45^,^46^, high accuracy classification can only be achieved by a model tolerant to these intrinsic variations. On the other hand, the feature extractor must reflect the intricate and tangled characteristics caused by extrinsic variations, eg. drug treatment^32^, cell cycles, rare cell types, labeling, and transcription rate^47^.[Fig f2]

**Figure 3:**
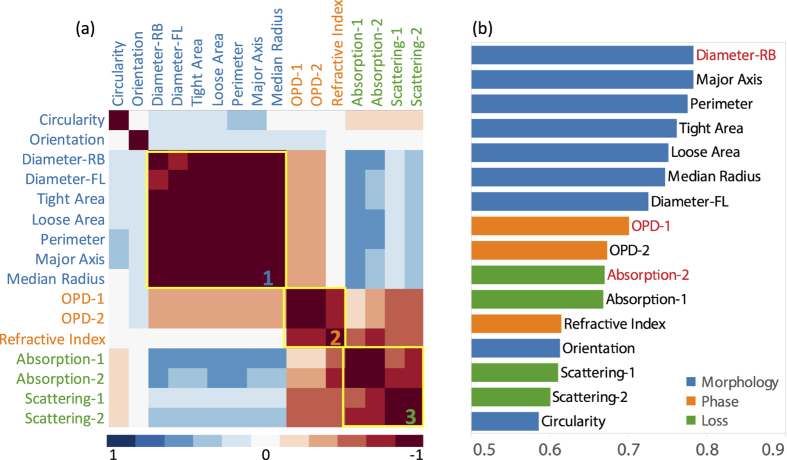
Biophysical features formed by image fusion. (**a**) Pairwise correlation matrix visualized as a heat map. The map depicts the correlation between all major 16 features extracted from the quantitative images. Diagonal elements of the matrix represent correlation of each parameter with itself, i.e. the autocorrelation. The subsets in box 1, box 2, and box 3 show high correlation because they are mainly related to morphological, optical phase, and optical loss feature categories, respectively. (**b**) Ranking of biophysical features based on their AUCs in single-feature classification. Blue bars show performance of the morphological parameters, which includes diameter along the interrogation rainbow, diameter along the flow direction, tight cell area, loose cell area, perimeter, circularity, major axis length, orientation, and median radius. As expected, morphology contains most information, but other biophysical features can contribute to improved performance of label-free cell classification. Orange bars show optical phase shift features i.e. optical path length differences and refractive index difference. Green bars show optical loss features representing scattering and absorption by the cell. The best performed feature in these three categories are marked in red.

A total of 16 features are chosen among the features extracted from fusion of optical phase and loss images of each cell. Features that are highly correlated do not provide unique information. Pairwise correlation matrix among these features is shown as a heat map in Fig. 3a. Diagonal elements of the matrix are correlation of each feature with itself, i.e. the autocorrelation. The subset of the features in Box 1 shows high correlation among morphological features. Also, the subset features in Box 2 and 3 are correlated as they are mainly related to optical phase shift and optical loss, respectively.

As a representation of our biophysical features in classification, Fig. 3b shows classification accuracy based on each single feature arranged in descending order. The features are color coded into three categories: morphology, optical phase, and optical loss, to describe the main type of information provided by each. The figure provides valuable insight into the relative importance of each category of cell features and suggests that morphological features carry the most information about cells, but at the same time, significant additional information is contained in optical phase and loss measurements.

### Machine Learning

**Figure 4:**
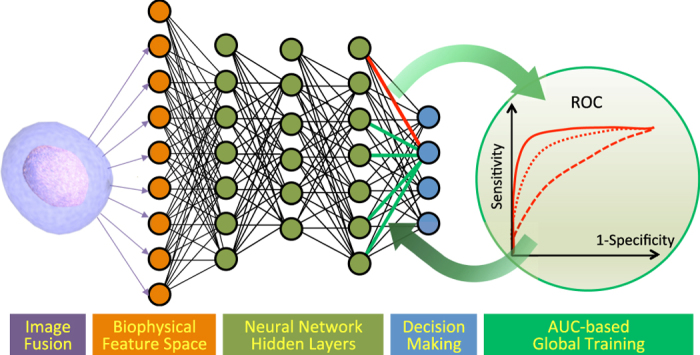
Machine learning pipeline. Information of quantitative optical phase and loss images are fused to extract multivariate biophysical features of each cell, which are fed into a fully-connected neural network. The neural network maps input features by a chain of weighted sum and nonlinear activation functions into learned feature space, convenient for classification. This deep neural network is globally trained via area under the curve (AUC) of the receiver operating characteristics (ROC). Each ROC curve corresponds to a set of weights for connections to an output node, generated by scanning the weight of the bias node. The training process maximizes AUC, pushing the ROC curve toward the upper left corner, which means improved sensitivity and specificity in classification.

Neural networks are a flexible and powerful bioinspired learning model, which perform layers of nonlinear feature transformations, learned from the training data^48^,^49^,^50^. The transformations morph the input data with weighted sums and nonlinear activation functions into feature spaces more suitable for classification. Shown in Fig. 4 is a unique feedforward neural network learning model that is globally trained by the objective of improving receiver operating characteristic (ROC). The learning algorithm introduced here maximizes the area under ROC curve (AUC), which is a global indicator of the classifier performance on the entire training dataset^51^,^52^,^53^. The global training of the neural network, although computationally costly, results in a classifier more robust, repeatable, and insensitive to imbalance among classes. For the purpose of end-to-end supervised learning with AUC whose gradient is not well-behaved, we employed the heuristic genetic algorithm (GA), which is resilient to discontinuities of the cost function and being trapped in local minima during optimization.

The network is composed of multiple hidden layers, which automatically learn representations of the data at different levels of abstraction, and thus, is considered a form of deep learning^54^,^55^. Each layer performs a linear combination on its inputs from the previous layer and operates a nonlinear function on the weighted sums. The output of the node *j* in layer 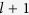, denoted by 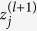 is generated from inputs *x*_1_^(*l*)^, *x*_2_^(*l*)^, …, *x*_*N*_^(*l*)^ as


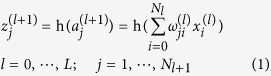


here 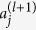 is the linear combination of inputs, and 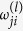 are the weights of the linear combination. The summation runs over 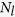, the total number of nodes in the layer *l*, and *L* is the total number of hidden layers. 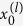, is the bias node in layer *l*, whose value is conventionally 1. Some popular choices for the nonlinear activation function 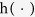 include logistic sigmoid function 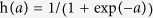, hyperbolic tangent function tanh(*a*), and commonly used in deep learning, rectified linear unit (ReLU) 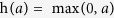. In our learning model, we use ReLU, which typically speeds up the supervised learning process of deep neural network by inducing sparsity and preventing gradient vanishing problem. The only exception is the output node, where we use the logistic sigmoid function as the activation function. The deep neural networks in our experiments have 3 hidden layers with 48 ReLUs in each.

For a trained classifier in hyperspace, receiver operating characteristics (ROC) curve describes the sensitivity and specificity of a classifier collection that includes nonlinear classifiers scaled in the direction of their normal vector field. In a deep learning network, this is equivalent to shifting the weight of the bias node in the last hidden layer. ROC highlights the trade-off between sensitivity and specificity (Fig. 4), and the area under ROC (AUC) provides a quantitative robust measure of classifier performance^56^,^57^,^58^,^59^. Choosing a large value for the weight of the bias node results in high sensitivity, but this sacrifices the specificity, leading to large number of false positives. As a way to visualize the impact of the threshold on classification accuracy, a classifier that accurately separates the classes will have an ROC curve that approaches the upper left corner. Conversely, a random guess, corresponding to balanced accuracy of 50% in binary classification will have an ROC that is a diagonal line. The AUC parameter serves as an effective analysis metric for finding the best classifier collection and has been proven to be advantageous over the mean square error for evaluating learning algorithms^60^.

To prevent overfitting in our deep learning model, we added a regularization term to the AUC-based cost function. Our regularization term is defined as mean square of all the network weights, excluding the weight of the bias nodes. Therefore, the overall cost function, 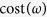, that is minimized by the genetic algorithm is


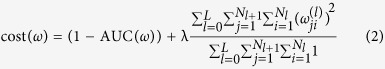


where λ is the regularization parameter, which controls the trade-off between overfitting (variance) and underfitting (bias).

### Demonstration in Classification of Cancerous Cells

**Figure 5:**
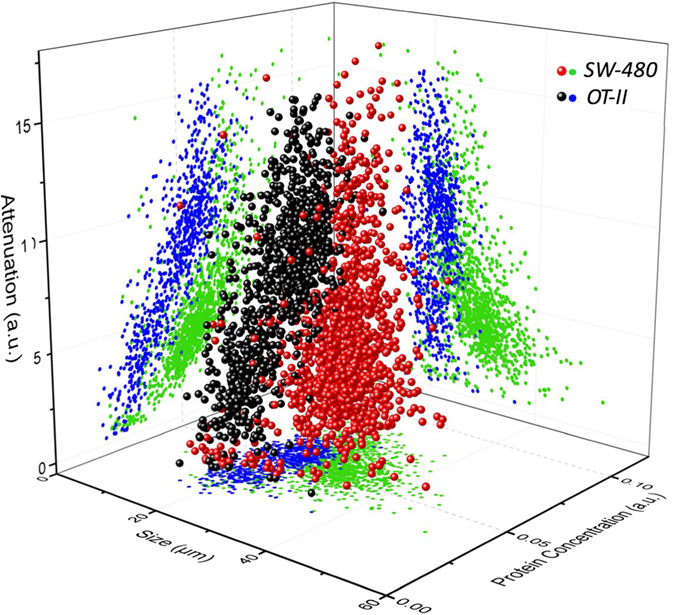
Three-dimensional scatter plot based on size, protein concentration, and attenuation of *OT-II* and *SW-480* cells measured by TS-QPI. The green and blue dots are two-dimensional (2-D) projections of cell data points on the planes containing only two of the biophysical features. The cell diameter along the rainbow (Diameter-RB) is used as a size indicator. The cell protein concentration corresponds to the mean refractive index difference of the cell (Refractive index feature in Table 1). The attenuation is a feature describing the optical intensity loss caused by cell absorption (Absorption-1 feature in Table 1). Comparison of 3-D and 2-D scatter plots reveals that additional biophysical features serve to classify the cell types more accurately.

In comparison with single-feature approaches^22^,^31^,^42^, our label-free cell classification enabled by TS-QPI and multivariate analysis, offers considerable improvements in detection sensitivity and accuracy for cancer diagnosis. To demonstrate the application in circulating tumor cell (CTC) detection, we used *OT-II* hybridoma T cells as a model for normal white blood cells and *SW-480* epithelial colon cancer cells. The features described in Table 1 were measured by our TS-QPI system for the aforementioned cells. Figure 5 shows three of these features in a three-dimensional (3D) scatter plot, attributed to size, protein concentration, and attenuation. The 2D projections on the three orthogonal planes are also shown. It is clear that additional dimensions improve distinguishment among different cell types compared to individual features.

**Figure 6:**
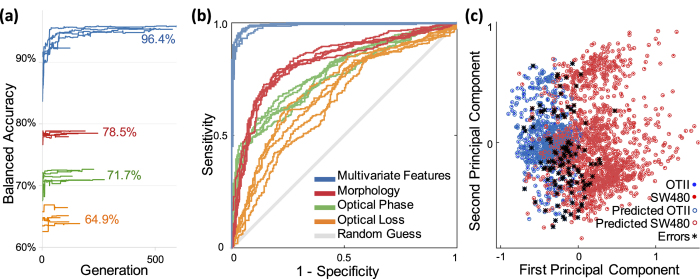
Classification of white blood cells (*OT-II*) and cancer cells (*SW-480*) by TS-QPI label-free features. (**a**) Training process of the neural network leads to improvement of classification accuracy over generations of genetic algorithm. In addition to multivariate analysis using all 16 biophysical features extracted from the TS-QPI quantitative images (blue curves), we also show training process by three single features. Red, green, and orange curves represent the best biophysical feature in each category, respectively: morphology (Diameter-RB in Table 1), optical phase (OPD-1 in Table 1), and optical loss (Absorption-2 in Table 1). The values represent average balanced accuracy among training datasets at the end of optimization. Clearly, the final achievable accuracy by multivariate classification is considerably higher than that of single features. (**b**) For each case, we show 5 ROC curves for different test datasets. The gray diagonal line shows results of random guess classification. Multivariate analysis based on TS-QPI images (blue curves) shows significant improvement in classification sensitivity and specificity. The fact that the classifiers remain almost unchanged during the five iterations of cross validation shows consistency and robustness of the classifiers. (**c**) To visualize the multivariate classification results, data points are depicted in the space of the first two PCA components.

A 5-fold cross-validation methodology is applied on the dataset to split data points into training, validation, and test subsets (details in supplementary material). Figure 6a shows progress in label-free classification depicted by balanced accuracy as the learning model evolves over GA generations. Blue curves show the classification balanced accuracy of the test dataset using all sixteen biophysical features extracted from the TS-QPI images. To highlight the improvement by hyperdimensional feature space of TS-QPI, we also show the balanced accuracy curves based on several single features: cell diameter for morphology, integral of cell’s optical path difference for optical phase information, and cellular absorption for optical loss in near-infrared window. Although these three biophysical features individually perform the highest accuracy among morphology, optical phase, and optical loss groups respectively, as previously shown in Fig. 3b, our multivariate deep learning classifier outperforms them. In addition, receiver operating characteristic (ROC) curves for each fold are generated based on the test subsets (Fig. 6b), and reveal the superior sensitivity and specificity of multivariate classifier. Also, the small variations of the ROC curves among different folds show the consistency of the classification performance for different test datasets. To visualize the hyperspace decision boundary, *OT-II* and *SW-480* data points are shown in first and second principal components analysis (PCA) components (Fig. 6c).

### Demonstration in Algal Lipid Content Classification

Microalgae are considered one of the most promising feedstock for biofuels^61^. The productivity of these photosynthetic microorganisms in converting carbon dioxide into useful carbon-rich lipids greatly exceeds that of agricultural crops. Worldwide, research and demonstration programs are being carried out to develop the technology needed to expand algal lipid production as a major industrial process. Selecting high-yield microalgae with fast growth factors are essential in biofuel production industry. Because algae differ greatly in size and structure, cell size alone provides insufficient information for cell classification. Here we show that adding optical phase and loss data, obtained by the phase contrast time stretch imaging flow cytometer, to size data enables algal cells to be distinguished on the basis of lipid content.

To test our apparatus for its ability to separate algal cells with high and low-lipid content, we exploited the starch-null *sta6* strain of *Chlamydomonas reinhardtii*. This strain is deleted for *sta6*^62^ (encoding the small subunit of ADP-glucose-pyrophosphorylase), and when nitrogen-deprived accumulates more lipid than wild-type^63^,^64^,^65^,^66^. Comparison of the two strains therefore provides an ideal setup to test our ability to distinguish lipid-content phenotypes.

**Figure 7:**
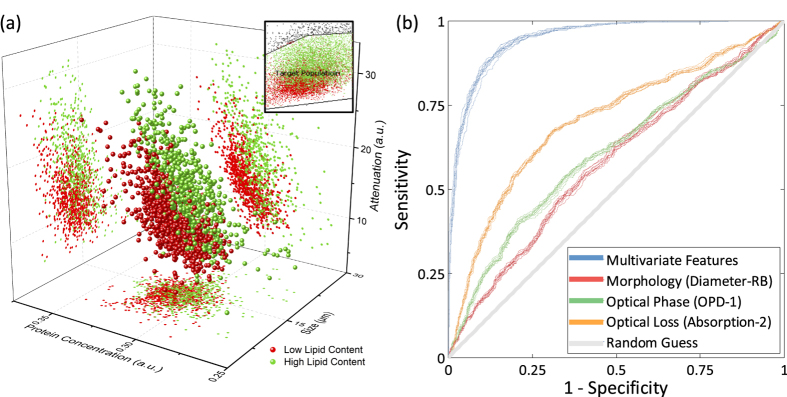
Classification of algal cells (*Chlamydomonas reinhardtii*) based on their lipid content by TS-QPI. (**a**) Three-dimensional scatter plot based on size, protein concentration, and attenuation of the cells measured by TS-QPI, with 2D projections for every combination of two features. Inset: Conventional label-free flow cytometry using forward scattering and side scattering is not enough to distinguish the difference between high-lipid content and low-lipid content algal cells. TS-QPI is much more effective in separating the two algae populations. (**b**) ROC curves for binary classification of normal and lipid-rich algae species using ten-fold cross validation; blue curves show the classifier performance using all 16 biophysical features extracted from the TS-QPI quantitative images. Red, green, and orange curves show the classifier decision performance using only the best biophysical feature in each category: morphology (Diameter-RB in Table 1), optical phase (OPD-1 in Table 1), and optical loss (Absorption-2 in Table 1). The label-free selection of algal strains improves as more biophysical features are employed.

Figure 7a shows the 3D scatter plot showing the three principal biophysical features for the two algal populations. Here, the optical loss category of the features plays a dominant role in label-free classification. In Fig. 7b, we show ROC curves for binary classification of these populations. Blue curves show the classifier performance using all 16 biophysical features extracted from the TS-QPI images. Red, green, and orange curves show the classifier decisions made using only the three major biophysical features: diameter for morphology (Diameter-RB in Table 1), optical path length difference for optical phase (OPD-1 in Table 1), and absorption for optical loss (Absorption-2 in Table 1). Our multivariate deep learning using TS-QPI is far more accurate than individual biophysical characteristics for selection of algal strains.

## Discussion

**Figure 8:**
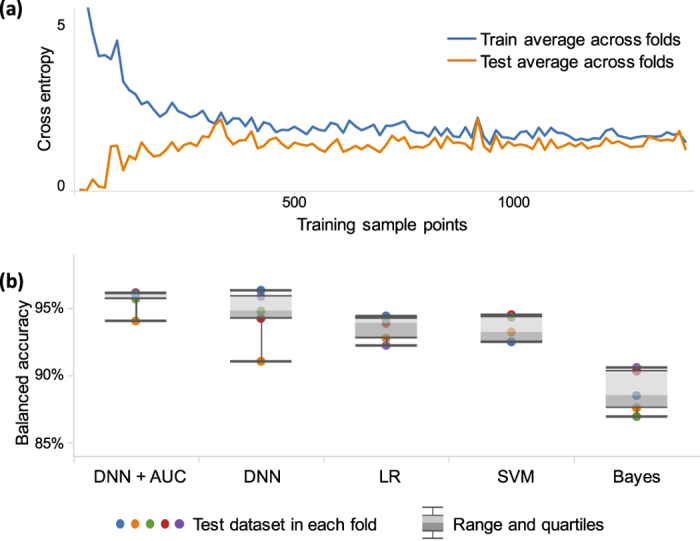
Learning curves and performance of various classification algorithms. (**a**) The learning curves of the training and test datasets in the tumor cell detection. Larger number of training data points decreases the cross entropy of the test dataset, which means the classifier is performing more accurately. However, the trend is opposite for the training dataset because the fitting error accumulates with a larger number of training data points. The discrepancy of the training and test errors, i.e. generalization error, decreases up to 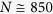, which is the necessary training data size for achieving final performance in our TS-QPI demonstration with deep learning neural network. (**b**) Comparison of multiple machine learning classification techniques based on the biophysical features extracted from the label-free cell images captured by TS-QPI. Our AUC-based deep learning model (DNN + AUC) has both the highest accuracy and consistency against support vector machine (SVM) with Gaussian kernel, logistic regression (LR), naive Bayes, and conventional deep neural network trained by cross entropy and backpropagation (DNN).

To show the effect of the training dataset size in the performance of the learning model, the learning curves for the training and test datasets of the tumor cell detection are analyzed (Fig. 8a). The test learning curve shows that as the number of training data points increases, the test error reduces and the model performance improves. On the other hand, the training error contrastingly increases for a larger number of training examples because it is more difficult for the learning model to fit many training data points than a few. The discrepancy of the training and test errors is the generalization error of the learning model^48^. Notice that beyond 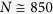 the generalization error do not decrease, and the learning curves converge to their ultimate performances. In other words, 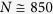 training data points are required to accomplish target achievable performance for the deep learning model used here.

Multiple machine learning techniques for multivariate label-free cell classification are compared using our TS-QPI tumor cell detection dataset (Fig. 8b). The mean accuracies of all learning models are beyond 85%, reflecting the advantages of simultaneous hyperdimensional biophysical features that TS-QPI provides for label-free cell classification. Furthermore, the interquartile range of the balanced accuracy (shown with box plot) is the smallest for the regularized AUC-based deep learning model, which confirms its consistency and repeatability are the best among learning methods.

## Conclusion

Time-stretch quantitative phase imaging (TS-QPI) is capable of capturing images of flowing cells with minimal motion distortion at unprecedented rates of 100,000 cells/s. TS-QPI relies on spectral multiplexing to capture simultaneously both phase and intensity quantitative images in a single measurement, generating a wealth of information of each individual cell and eliminating the need for labeling with biomarkers. Here, we summarized the information content of these images in a set of 16 features for each cell, and performed classification in the hyperdimensional space composed of these features. We demonstrated application of various learning algorithms including deep neural networks, support vector machine, logistic regression, naive Bayes, as well as a new training method based on area under the ROC curve. The results from two experimental demonstrations, one on detection of cancerous cells among white blood cells, and another one on identification of lipid-rich algae, show that classification accuracy by using the TS-QPI hyperdimensional space is more than 17% better than the conventional size-based techniques. Our system paves the way to cellular phenotypic analysis as well as data-driven diagnostics, and thus, is a valuable tool for high-throughput label-free cell screening in medical, biotechnological, and research applications.

## Methods

### Time Stretch Quantitative Phase Imaging (TS-QPI) System

Broadband optical pulses from a mode-locked laser (center wavelength = 1565 nm, repetition rate = 36.6 MHz, pulse width 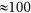 fs) are broadened using a highly nonlinear fiber to approximately 100 nm bandwidth with a spectral range up to 1605 nm. These broadband pulses are then linearly chirped to nanosecond pulse width by a short dispersion compensating fiber (DCF) of 60 ps/nm, so that an erbium doped fiber amplifier (EDFA) can amplify them with minimal distortion. A coarse wavelength division multiplexer (WDM) filters the pulses from 1581 nm to 1601 nm, where the spectrum is reasonably flat. Therefore, the total bandwidth of the pulses interrogating the cells in our setup is less than 20 nm centered at 1591 nm, giving a negligible fractional bandwidth of 1.3%. These filtered pulses then pass through an optical circulator and are coupled to free-space with a fiber collimator.

Free-space laser pulses were linearly polarized with quarter- and half-wave plates, and then spatially dispersed with a pair of reflection diffraction gratings, so that each wavelength component of the collimated beam was positioned at a different lateral point similar to a line flash rainbow. A beam reducer shrank the rainbow beam 6 times with a pair of 90 degree off-axis parabolic gold-coated mirrors with reflected focal lengths of 152.4 mm and 25.4 mm, respectively. Next, a 15 degree off-axis parabolic gold-coated mirror with 635 mm reflected focal length and a long working-distance objective lens with 0.4 numerical aperture further shrank the rainbow to about 130 in width, i.e. field of view. Reflective optics with parabolic gold-coated mirrors is used in our experimental demonstration to minimize loss, aberration, and polarization sensitivity. The rainbow flashes were then split into the two arms of a Michelson interferometer by a beam splitter. In the sample arm, the rainbow pulses pass through the cells and are reflected by the reflective substrate of the microfluidic device. In the reference arm, a dielectric mirror reflected the rainbow with a length mismatch with the sample arm causing spectral interference fringes (Fig. S1a). Cells are hydrodynamically focused at the center of the channel flow at a velocity of 1.3 m/s. The reflected pulses from reference and sample arms were recombined at the beam splitter, compressed by the two diffraction gratings and coupled back into the fiber. These return pulses were spectrally encoded by the spatial information of the interrogation field of view. Then they were redirected by the optical circulator to a Raman-amplified time-stretch dispersive Fourier Transform (TS-DFT) system followed by a 10 Gb/s photodetector (Discovery Semiconductors DSC-402APD). An analog-to-digital converter (Tektronix DPO72004C) with a sampling rate of 50 GS/s and 20 GHz bandwidth is used to acquire the output signal of the photodetector, which is a series of spectral interferograms mapped into time (Fig. S1b).

### Coherent Detection and Phase Extraction

Unlike conventional heterodyne detection, which uses a narrowband continuous-wave signal as the local oscillator or reference, the coherent detection in our time stretch system uses an unmodulated copy of the original optical input, which is a broadband optical pulse train^67^,^68^.

Since the spectrum is mapped into space by diffraction gratings, the complex field at any specific spatial location within the field of view is a narrowband optical wave. As the envelope of the optical wave varies slowly in time compared to the period of the optical electromagnetic wave and the time mismatch between the reference arm and the sample arm, we employ slowly varying envelope approximation in our analysis. The complex envelope of the input electric field, 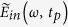, is split into two arms of the Michelson interferometer at the beam splitter. Here, *ω* is the optical frequency of the input signal, which corresponds to the spatial location *x* being interrogated by the optical wave at this frequency (i.e. spectral encoding of the object image). 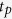 specifies the time when each rainbow flash reaches the beam splitter, corresponding to the *p*-th incoming pulse. Note that 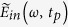 can be simplified as 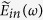 when pulse shape is stable from pulse to pulse. The light split into the two arms of the Michelson interferometer can be expressed as

Into the sample arm:


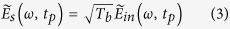


Into the reference arm:


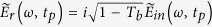


where 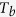 is the power transmission ratio of the beam-splitter. Optical intensity in the sample arm will be altered by the absorption and scattering of imaged cells, as well as that of the microfluidic channel and buffer solution. Not only the electric field amplitude after passing through semitransparent objects will be modulated by the optical attenuation in the sample arm, but also the optical path length difference will lead to a phase shift, 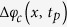, induced by refractive index change caused by the object along the interrogation beam. Thus, the complex fields of the light waves coming back to the beam splitter become

From the sample arm:


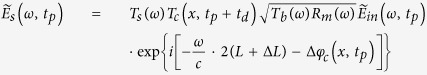


From the reference arm:


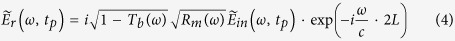


where *L* is the length of reference arm, and 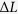 is the arm length mismatch between two arms. 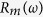 is the wavelength-dependent reflectance of the reflective substrate of the microfluidic channel and the dielectric mirror in the reference arm. 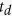 is the time delay during which rainbow flash travels from the beam splitter to the sample cell, 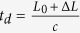. 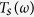 is power transmittance of the surrounding buffer solution and microfluidic channel, and 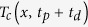 is spatial power transmittance of cells at location *x* along the rainbow when being illuminated at time 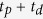. Both 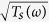 and 
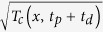
 affect the optical field twice as each rainbow flash passes through the cell twice. Since the 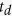 is much smaller than the time scale of the envelope variations caused by the cell flow, we can approximate 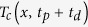 to be 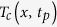 without sacrificing accuracy.

After the pulses from two arms of the interferometer recombine at the beam splitter, the total electric field at each wavelength or optical frequency becomes


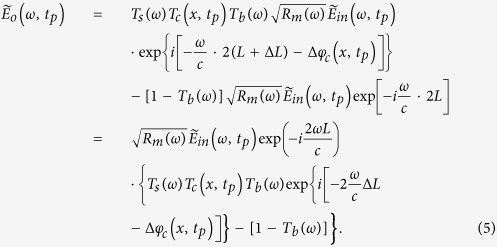


Based on the spectral encoding setup, we know spatial information has been encoded into spectrum,


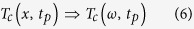



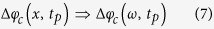


the intensity envelope then becomes


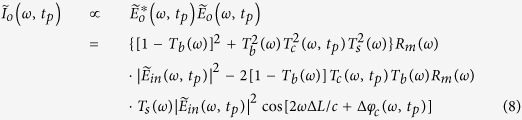


During time stretch, each frequency component *ω*, or wavelength λ will be one-to-one mapped into time domain. We define the relative time delay of λ compared to the central wavelength, 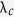, as 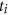, which is usually called intra-pulse time delay. Written in terms of λ, Eq. 8 can be simplified as


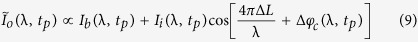


where 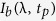 is the background or baseband intensity envelope, and 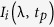 is the interference or intermediate intensity envelope:


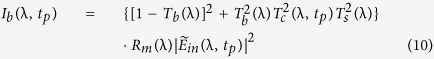



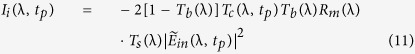


The linear time stretch maps frequency domain into time domain by


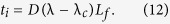


Here 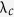 is the central wavelength and 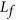 the length of the dispersive fiber. *D* is the group velocity dispersion, that is, the temporal pulse spreading, 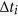, per unit bandwidth, per unit distance traveled. Thus the temporal samples of the energy flux absorbed at the photodetector are the intra-pulse concatenation of spectral samples followed by inter-pulse concatenation of pulse waveforms:


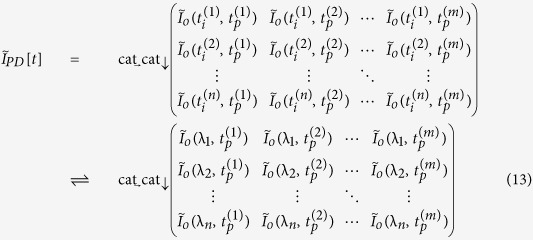


where 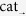 and 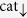 mean horizontal and vertical concatenations, respectively. Each 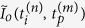 expresses the 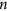
th spectral (spatial) pixel at the 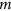
th pulse (line image). Applying Eq. 12 to Eq. 9,


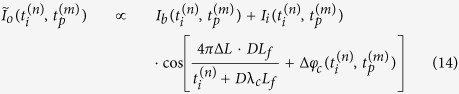


Therefore, the time stretched temporal waveform corresponding to each line scan image consists of two features^20^. One is 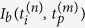, a temporal envelope of the time-stretched optical pulse at baseband frequencies. The amplitude of this envelope corresponds to the temporal shape of the optical pulse and its deviations caused by the object transmission as in brightfield microscopy. It provides information about optical loss, i.e. light absorption and scattering caused by surface roughness, granularity, and inner cell organelle complexity.

The second term in Eq. 14 (with cosine component) is a fast oscillating fringe, caused by the spectral interference of the recombined pulses from the sample and the reference arms in the Michelson interferometer. This term can be separated by a bandpass filter, and its envelope can be derived by a nonlinear envelope detection technique. Here we used a moving minimum/maximum filter to extract the envelope. After normalization to the envelope, the cosine component


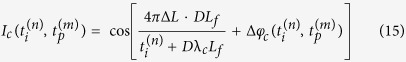


is used for calculation of the object phase shift, 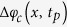. The first term in cosine causes the interferogram fringe pattern. Since 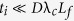, it can be approximated as


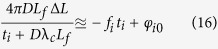


where 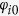 is an initial phase constant, 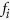 is the fringe frequency:


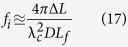


As seen in Fig. S1b, the fringe frequency, 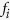, in our setup is about 4.7 GHz determined by the optical path length mismatch between the interferometer arms.

The instantaneous phase of 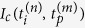 can be readily retrieved from its analytic representation given by Hilbert transform, 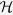:


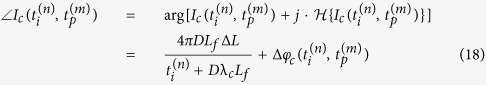


here arg means the argument of a complex number. A one-dimensional phase unwrapping algorithm followed by background phase removal gives the object phase shift,


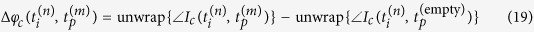


where 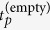 corresponds to an empty pulse when no cell is in the field of view, i.e. background phase. The unwrapping algorithm used in our processing acts when the absolute phase difference between two consecutive samples of the signal is greater than or equal to *π* radians, and adds multiples of 2*π* to the following samples in order to bring the consecutive sample phase differences in the acceptable range of −*π* to *π*.

To perform combined quantitative phase and loss imaging, the phase derived by Hilbert transformation should be corrected to eliminate the artifacts caused by the intensity variations induced by the passing cells. Most cells of interest in clinical or industrial applications have a diameter 3–40 μm, when suspended in fluid. Given the field of view and the period of the interrogation rainbow pulses are 130 μm and 27 ns, respectively, the time duration of the instantaneous intensity change induced by the single cells in each laser pulse is about 0.6–8.3 ns, which will generate baseband frequency components up to about 1.6 GHz. Compared to the higher frequency components at 4.7 GHz corresponding to the interference fringes, the frequency of intensity variations is small (<1.6 GHz), and in this scenario, our method remains robust to separate the two electrical spectral components for optical loss and phase.

### Cell Transmittance Extraction

One of the advantages of TS-QPI is its ability to extract the cell transmittance, *T_c_*(λ)without prior knowledge of the transmittance of the solution, 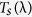, that of the beam-splitter, 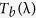, and the reflectance of substrate of the microfluidic channel, 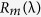. During measurements when there is no cell in the field of view (empty frames), Eq. 11 becomes


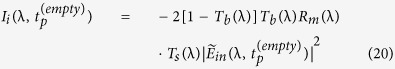


In addition, the signal from only the reference arm can be recorded by blocking the sample arm:


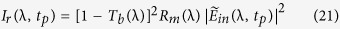


Combining Eqs 10, 20, and 21, and assuming that the input electric field pulse shape, 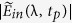, is invariant to 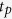, the cell transmittance can be derived as


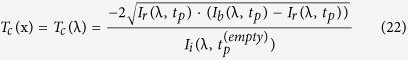


Please note that the values of 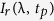, 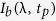, and 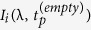 are directly measured by TS-QPI, and no prior knowledge of 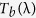, 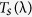, 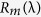, and 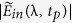 is needed to calculate the cell transmittance.

### Image Reconstruction

We reconstruct both quantitative brightfield and phase-contrast images simultaneously from single-shot frequency-multiplexed interferometric measurements. The envelope and phase of the time-domain signal 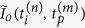 was firstly mapped into series of spatial information 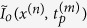, forming linescan bright-field and phase-contrast images, illuminated by the optical pulse at time 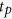. This is because within each optical pulse, the spatial information is mapped one-to-one into spectral domain, 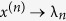, and spectrum is stretched in time, 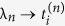, where 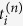 is the relative group delay time of each frequency component within a pulse with respect to the central wavelength. These line-scan images based on 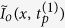, 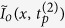, 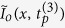, … were then cascaded into a two dimensional image corresponding to 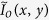, where the second dimension *y* is the spatial mapping of time lapse based on object flow speed.

The optical path length difference image can be calculated by the phase shift line scans as


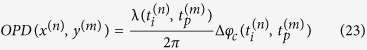


On the other hand, if the axial thickness of the cell at reconstructed image pixel 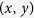 is 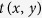,


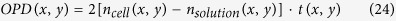


in which 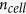 and 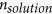 are the refractive indices of the cell and the surrounding buffer solution, respectively. The factor 2 is to account for the fact that each wavelength component passes the cell twice in Michelson interferometer.

If we integrate Eq. 24 over the area of the cell, we can derive an average refractive index contrast for the cell, which corresponds to the average protein concentration of the cell:


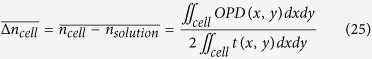


where 
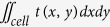
 is the volume of the cell obtained from its lateral diameter, *d*, as 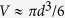.

The relative net change of intensity envelope variations induced by the cell is obtained from the amplitude of the baseband intensity envelope of the interferogram as


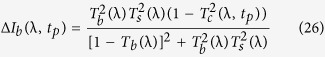


It gives the temporal and spatial information of the combined effects from absorption and scattering:


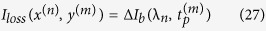


### Big Data Analytics Pipeline

The high-content image analysis and cell screening pipeline is implemented by combining multiple informatics tools, namely CellProfiler for image processing^6^,^68^, MySQL/MangoDB for database, Matlab for machine learning, and Javascript for interactive visualization. First of all, image noise reduction and smoothing have been performed, which can remove artifacts that are smaller than optical resolution limit. For object segmentation, we use the Otsu’s thresholding method. Once objects are identified in the image, morphology of each single cell can be described by area, diameter, uniformity, aspect ratio, perimeter, number of surrounding clumped cells, etc.

The capability to identify clumped cells from single large cells greatly reduces the misclassification rate in imaging flow cytometry compared to traditional flow cytometry. Intensity peaks of pixel brightness within each object are used to distinguish clumped objects. The object centers are defined as local intensity maxima in the smoothed image. Retaining outlines of the identified objects helps validate and visualize the algorithm. In the next step, we discard the objects touching the borders of the image, i.e., the edges of the field of view and data acquisition time window. However, the chance of cells showing up at the edges is very low due to hydrodynamic focusing. We are also capable of excluding dust, noise, and debris by neglecting the objects that are too small or have extreme aspect ratios.

To calibrate the imaging system and image processing pipelines for size measurement, 5 μm polystyrene beads (from Polysciences, Inc.) with National Institute of Standards and Technology (NIST) traceable particle size standards were analyzed. Size measurement of the polystyrene beads had a distribution with 5.06 μm expected mean and 0.5 μm standard deviation. The broadened standard deviation was within the range of optical resolution limit and was caused mainly by performing object recognition on resolution limited images. Due to limited optical resolution of the setup, the edges of bead or cell are blurred, generating distribution of point spread functions in optical phase and loss images outside of the cell boundaries. In order to maximize the accuracy in morphological, phase, and loss measurements, after object segmentation we expanded the object boundaries by 2.5 μm (optical resolution of the setup measured by knife-edge method), which serve as loose boundaries, indicating the area within which the pixel intensities are measured and integrated in phase and loss images.

### Data Cleaning

Data cleaning includes two steps. Firstly, Hotelling’s T-squared distribution is calculated and the top 2% of the extreme data was set as outliers due to experimental or object recognition errors. Secondly, debris discrimination is performed; any data point with negative phase shift was considered as either air bubble, flow turbulence, or object recognition errors.

## Additional Information

**How to cite this article**: Chen, C. L. *et al.* Deep Learning in Label-free Cell Classification. *Sci. Rep.* **6**, 21471; doi: 10.1038/srep21471 (2016).

## Supplementary Material

Supplementary Information

Supplementary Video 1

Supplementary Video 2

## Figures and Tables

**Table 1:** List of extracted features.

Feature Name	Description	Category
Diameter-RB	Diameter along the interrogation rainbow. It is insensitive to flow rate fluctuation. For higher accuracy, it is calibrated by the spatial nonuniform distribution of rainbow wavelengths.	Morphology
Diameter-FL	Diameter along the flow direction. It is sensitive to flow rate fluctuation, but can be a candidate parameter for monitoring flow speed and channel condition.	Morphology
Tight Area	Total number of pixels in the segmented region in the phase image.	Morphology
Perimeter	Total number of pixels around the boundary of each segmented region.	Morphology
Circularity	4*π* (Tight Area)/Perimeter^2^	Morphology
Major Axis	Considering the cell as elliptical in lateral imaging plane, the length of the major axis of the ellipse with a normalized second central moment same as the cell.	Morphology
Orientation	Angle between the flow direction and the major axis of the cell elliptical shape.	Morphology
Loose Area	Total number of pixels in the expanded segmented region for measurement of the pixel intensities.	Morphology
Median Radius	The median distance of any pixel in the object to the closest pixel outside of the object.	Morphology
OPD-1	Integrated optical path length difference within the entire segmented area (cell), calibrated by the power distribution within different wavelength components of the incident laser pulses.	Optical Phase
OPD-2	Integrated optical path length difference within the entire segmented area (cell). In addition to the calibration of OPD-1, it is calibrated by the pulse-to-pulse fluctuations within a 1µs detection window.	Optical Phase
Refractive index	The mean refractive index difference between the object and the surrounding liquid (buffer solution), which is calculated based on OPD-2 and size measurement (see detail in Section Methods). Refractive index difference for cells is proportional to their protein concentration.	Optical Phase
Absorption-1	Mean absorption coefficient within the entire segmented area (cell). It is calibrated by the power distribution within different wavelength components of the incident laser pulses and by the pulse-to-pulse fluctuations within a 1µs detection window. This parameter corresponds to an absorption-dominant model for the cell.	Optical Loss
Absorption-2	Mean absolute absorption coefficient within the entire segmented area (cell). It is calibrated by the power distribution within different wavelength components of the incident laser pulses and by the pulse-to-pulse fluctuations within a 1µs detection window. This parameter corresponds to an absorption-dominant model for the cell.	Optical Loss
Scattering-1	Mean optical loss within the entire segmented area (cell). It is calibrated by the power distribution within different wavelength components of the incident laser pulses and by the pulse-to-pulse fluctuations within a 1 detection window. This parameter corresponds to a scattering-dominant model for the cell.	Optical Loss
Scattering-2	Mean absolute optical loss within the entire segmented area (cell). It is calibrated by the power distribution within different wavelength components of the incident laser pulses and by the pulse-to-pulse fluctuations within a 1µs detection window. This parameter corresponds to a scattering-dominant model for the cell.	Optical Loss
