# Supplementary material for: Deep Learning in Label-free Cell Classification
Source: Sci Rep. 2016 Mar 15;6:21471. doi: 10.1038/srep21471 (PMC4791545; doi:10.1038/srep21471)
Supplement: Supplementary Information [file srep21471-s1.pdf]

Deep Learning in Label-free Cell Classification

Claire L. Chen, Ata Mahjoubfar, Li-Chia Tai, Ian K. Blaby, Allen Huang, Kayvan R. Niazi, and Bahram Jalali
(Dated: November 21, 2015)

SUPPLEMENTARY METHODS

PRINCIPAL COMPONENT ANALYSIS (PCA)

As shown in Fig. 3a and Fig. 3b, many of the 16 features are correlated and not all measured features in the data set produced by the time stretch quantitative phase imaging have the same amount of information. That result suggests that it may be possible to reduce the 16 dimensional data set to a smaller set of uncorrelated orthogonal dimensions without significantly compromising the classification accuracy. In that spirit, we have used principle component analysis (PCA) for dimensionality reduction and computation speed-up. The PCA algorithm finds an alternative lower dimension space such that variance of data projected onto this subspace is maximized along subspace dimensions. Fig. S2a shows the percent of the variance in data explained by each component (lower chart). The key observation is that most of the variance can be accounted for by the first two principle components. The upper portion of the plot shows the accuracy for binary classification using each of the principle components. Interestingly, the first component with the highest explained variance is not necessarily the most important component for classification. Therefore, apriori intuition about the physical significance of the features in the case here, is superior to PCA in eliminating dimensions that do not provide high value in classification. By revealing the structure in data that best explains the variance, PCA achieves data compression via dimensionality reduction.

PCA components acts as the input features for the classification algorithm. As number of PCA components retained increases, the classification accuracy improves while computation time increases (Fig. S2b). Since accuracy is the main concern here, we employ all 16 biophysical features, rather than dimensionality-reduced PCA components.

CROSS VALIDATION

The k-fold cross-validation implemented here splits data points into training, validation, and test subsets (Fig. S3a). For each iteration, one fold is used as test data, one for validation while the other folds are used during training process. After initially trained, the performance of the network is analyzed by the validation data to fine tune the neural network architecture and regularization parameter. Fig. S3b shows that either a too small or a too large regularization parameter, λ, increases network error due to overfitting or underfitting, respectively. Therefore, there is a suitable range of regularization parameter for each learning model.

Once the network architecture and regularization parameter are chosen and optimized based on the validation data, the learning model performance is finally verified by the test fold, which has never been used before in this iteration. The process of training in each iterations is independent, so each iteration has no prior knowledge about the chosen learning models in other iterations. The final reported results are aggregate of the performance for different test datasets.

COMPUTATION TIME

Our deep learning technique uses AUC as the cost function and performs training via genetic algorithm. Since AUC is calculated based on the entire dataset, the genetic algorithm is employed as a global optimization method. Thus, our technique has inherently higher accuracy and repeatability compared to conventional deep learning and other classification algorithms studied here. However, the global optimization in our algorithm sacrifices the computation time. The performance of balanced accuracy and computation time of different classification algorithms are compared in Table S1.

SYSTEM PERFORMANCE AND RESOLVABLE POINTS

Lateral resolution of time stretch camera is decided by the limiting factor among Abbe diffraction limit of the objective lens, spectral resolvability of the diffraction grating pairs, spectral resolution in amplified dispersive Fourier transform, the photodetector rise-time and bandwidth, and the sampling rate of the back-end digitizer. Details of the limiting factors of lateral resolution and evaluation of these factors for our TS-QPI system can be found in Table S2. Field of view (FOV) is the area covered by the interrogation rainbow when the rainbow pulses hit the imaging plane. The rainbow pulse width is decided by the optical bandwidth selected from the laser source, $\Delta\lambda$, the magnification factor of the objective lens, the focal length of the other lenses and parabolic mirrors, as well as the dimensions and blaze angles of the diffraction gratings.

The resolution of phase measurement along axial direction is determined by the effective number of bits (ENOB) of the digitizer and affected by the noise of laser source. Since pulse-to-pulse intensity and phase fluctuations are small, noise from laser source is not the limiting

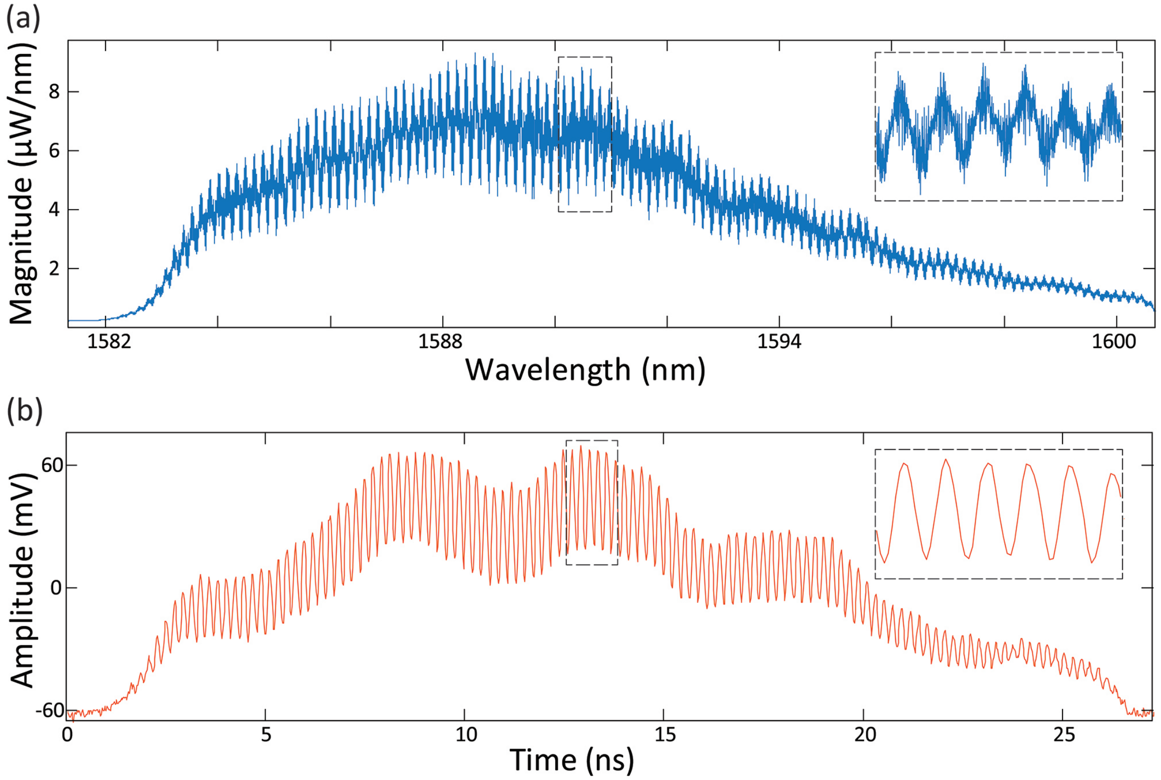

FIG. S1. Comparison of the interferograms measured by optical spectrum analyzer and time-stretch dispersive Fourier Transform; (a) Optical spectrum of the signal after quantitative phase imaging (box 1 in Fig. 1) and before it enters the amplified time-stretch system (box 2 in Fig. 1). The interference pattern in spectral domain is measured by an optical spectrum analyzer. (b) With time stretch, the interference pattern in spectral domain is linearly mapped into time. The baseband intensity envelope is slightly modified by the wavelength-dependent gain profile of the Raman amplifier. The inserts in panels a and b show the zoomed-in spectrum and waveform in the dashed black boxes, respectively. Clearly, the single-shot interferogram measured by Raman-amplified time-stretch dispersive Fourier Transform has a higher signal-to-noise ratio compared to that captured by optical spectrum analyzer.

TABLE S1. Performance comparison of different classification algorithms

Algorithm	Averaged balanced accuracy	Standard deviation of balanced accuracy	Computation time (second)
Deep neural network trained by AUC	95.5%	0.9%	365.6
Deep neural network trained by cross entropy	94.4%	2.1%	4.7
Logistic regression	93.5%	0.9%	0.8
Support vector machine	93.4%	1.0%	1.7
Naive Bayes	88.7%	1.6%	2.8

factor in our phase measurements. Supposing the ENOB of the digitizer is N, the minimum detectable optical path length difference, ΔL can be estimated as

$$\frac{1}{2} sin\left(\frac{4\pi\Delta L}{\lambda+\Delta\lambda/2}\right) = 2^{-N} \qquad (6)$$

where λ is the central wavelength of light, and $\Delta\lambda$ is the optical bandwidth. In our system, ENOB of the analog-to-digital converter is 5. Thus, the OPD resolution along the axial direction is about 8.0 nm, corresponding to refractive index difference down to the order of 0.001 for cellular level measurements.

MICROFLUIDIC CHANNEL DESIGN AND FABRICATION

The Polydimethylsiloxane (PDMS) microfluidic channel is custom-designed so that it could fit into the reflective optics design. Cells are hydrodynamically focused [1, 2] at the center of the channel flowing at a velocity of 1.3 m/s. The microfluidic device consists of a hydrodynamic focusing region and an imaging region targeted by the interrogation rainbow flashes in TS-QPI system. At the hydrodynamic focusing region, the sheath pressure focused the sample at the center of the channel by narrowing its flow width from 200 μm to about 40 μm with

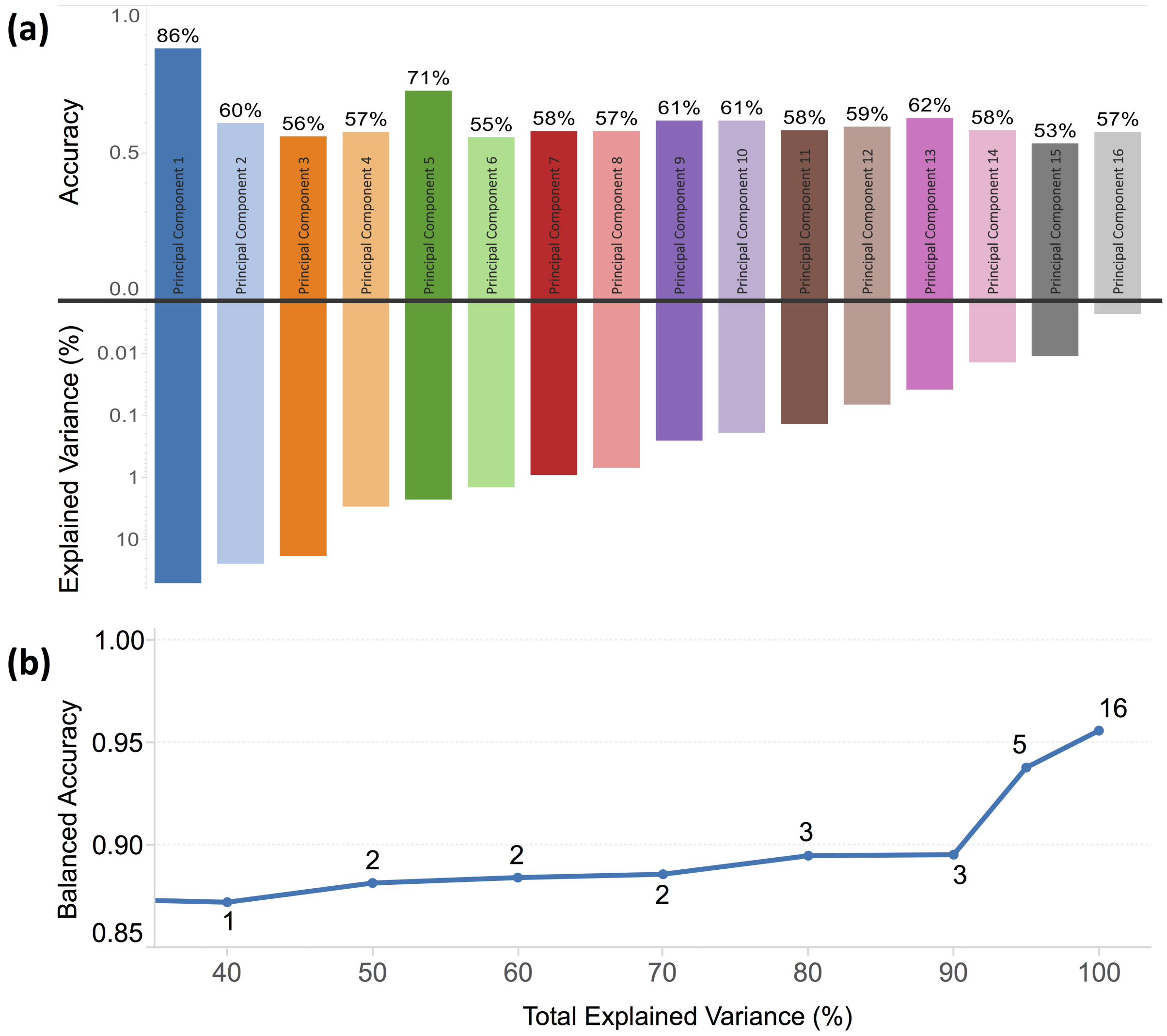

FIG. S2. Principal component analysis (PCA) on the multivariate data set produced by time stretch quantitative phase imaging. (a) Upper bar chart shows accuracy of classification by each individual principal component, and lower bar chart shows the percentage of the total variance explained by each principal component, accounting for the variability expressed in the data. As expected, principal components with larger variability do not necessarily give high accuracy in classification. (b) Cumulative accuracy. The value at each data point corresponds to the number of PCA components retained in order to achieve that total explained variance. In order to reduce the number of input features and decrease computation time, a subset of the PCA components can be used for classification. The classification accuracy improves as the total variance retained in the subset of PCA components goes up. Nearly 90% accuracy can be achieve with the first three PCA components. The small deviation among accuracies of data points with the same number of PCA components are due to variations in random data partitioning.

a sheath to sample volume ratio of 3:1. The dimension of the channel was chosen as 200 µm (width) × 25 µm (height) so that the cells will be imaged within depth of focus with a narrow lateral distribution. The size of the entire PDMS channel is optimized for fitting on a 2 inch diameter dielectric mirror with sufficient space at the edges to achieve strong bonding. The thickness of the channel top layer is optimized for stabilizing peek tubes performance reliability while accommodating the working distance of the objective lens.

The PDMS microfluidic channel (Fig. S4) is fabricated using standard soft lithography. The mask was designed in AutoCAD and printed with a resolution down to 1 µm. Then a 4-inch silicon wafer was spin-coated with 75 µm thickness of a negative photoresist (SU-8 from MicroChem) and was exposed under the mask using an aligner. After post-exposure baking, the wafer was developed at room temperature, rinsed with isopropyl alcohol (IPA), and placed in a petri dish. A PDMS mixture (Sylgard 184 Silicone Elastomer, Dow Corning) was poured onto the patterned wafer, degassed in a vacuum chamber for 30 min and cured at 80 °C for one hour. Once cured, the PDMS channel was cut out and peeled off from the master wafer. We used 1.25 µm diameter hollow needle

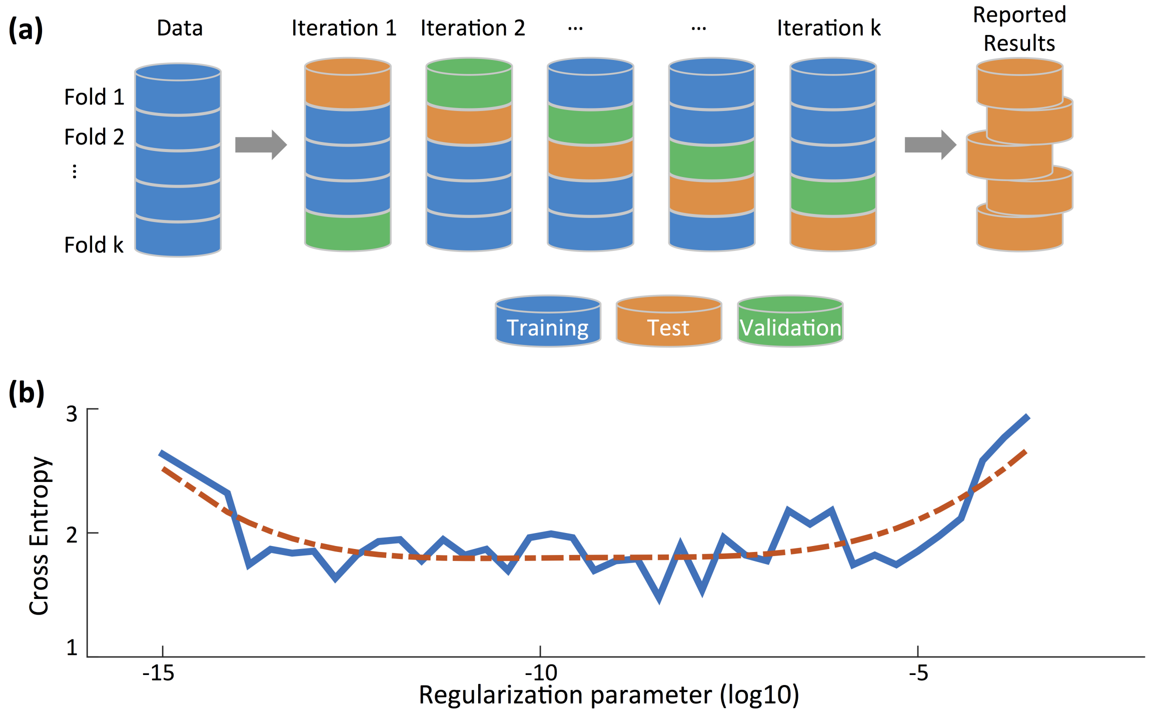

FIG. S3. (a) The implementation of the k-fold cross-validation here splits data points into training, validation, and test subsets. In each iteration, one fold is used for fine tuning the learning model (validation dataset) and another fold is used for evaluation of the final results (test dataset), while rest of the data points are used for training (training dataset). The final reported results are aggregate of the outcomes from the test datasets. (b) A suitable regularization parameter, λ, balances the trade-off between overfitting (variance) and underfitting (bias) and minimizes the cross entropy of the validation dataset.

TABLE S2. Resolution Limiting Factors in TS-QPI

System catagory	Component	Number of resolvable points	Lateral resolution
Free-space optics	Diffraction gratings	$N_{grating} = \frac{\Delta\lambda}{\delta\lambda_{grating}} = \Delta\lambda/(\lambda \cdot \frac{d}{m \cdot 2w_0})$ (1) where $\Delta\lambda$ is the optical bandwidth, λ is the central wavelength, m is the order of diffraction, w_0 is the beam waist, and d is the groove spacing.	3.09 µm
	Lenses and mirrors	$N_{Abbe} = \frac{FOV}{\delta x_{diffraction}} = \frac{FOV}{(\frac{\lambda+\Delta\lambda/2}{2 \cdot NA})}$ (2) where FOV is field of view, NA is numerical aperture of the objective lens.	2.00 µm
Time Stretch	Group delay dispersion	$N_{DFT} = \frac{\Delta\lambda}{\delta\lambda} = \frac{\Delta\lambda}{\lambda \cdot \sqrt{\frac{2}{DL_f \cdot c}}}$ (3) where D is the group velocity dispersion, L_f is the dispersive fiber length.	0.73 µm
Electronic back-end	Photodetector bandwidth	$N_{PD} = \frac{\Delta t}{\delta t} = \frac{DL_f\Delta\lambda}{0.35/B}$ (4) where B is the bandwidth of the photodetector.	0.28 µm
	ADC sampling rate	$N_{ADC} = DL_f\Delta\lambda f_{ADC}$ (5) where f_{ADC} is the sampling rate of digitizer.	0.10 µm

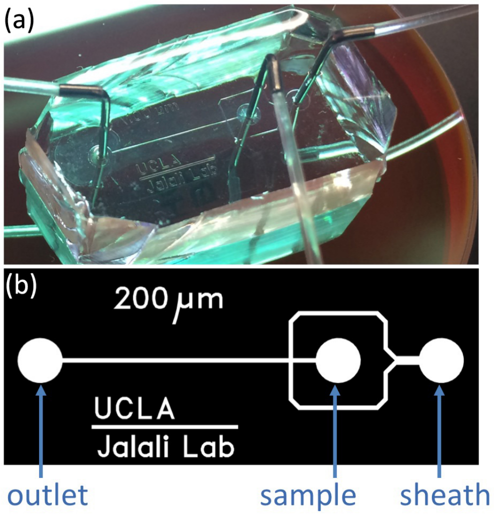

FIG. S4. PDMS microfluidic channel mounted on a highly reflective surface with near-infrared dielectric coating; The microfluidic device consists of a hydrodynamic focusing region and an imaging region targeted by the interrogation rainbow flashes in TS-QPI system. (a) Sample solution with suspended cells is fed into the channel through the sample inlet, and deionized water as the sheath fluid is injected through the sheath inlet. At the hydrodynamic focusing region, the sheath pressure focused the sample at the center of the channel by narrowing its flow width from 200 µm to about 40 µm with a sheath to sample volume ratio of 3:1. (b) The pattern of the mask used to imprint microfluidic channel design on silicon wafer with photoresist. The circles are inlet and outlet reservoirs.

to punch the inlet and outlet holes. The punched PDMS channel was then cleaned with nitrogen gun and magic tape (3M), treated with oxygen plasma (Enercon Dyne-A-Mite 3D Treater) for 2 min, and bonded to a 2-inch diameter broadband dielectric mirror (Thorlabs BB2-E04) for obtaining high reflectance from channel substrate at near infrared spectral window. Finally microtubes (PE-50 tubing, .023 $\times$ .038 in) with steel catheter couplers (Instech, 22 ga $\times$15 mm) are connected to the inlet and outlet punctures.

PREPARATION OF ALGAE CELL LINES

Chlamydomonas reinhardtii strains used were *cw15* (*nit1 NIT2 mt*$^{+-}$) and *sta6* (*cw15 nit1 NIT2 arg7-7 sta6-1::ARG7 mt*$^{+}$), available as CC-4568, CC-4348 respectively from the Chlamydomonas resource center (CRC)[3].

Cells were grown in tris-acetate-phosphate (TAP) medium supplemented with arginine (100 µg mL^{-1}). Cultures were grown in Innova incubators (New Brunswick Scientific, Edison, NJ) at 24 °C, agitated at 180 rpm with continuous light (95 µmol m^{-2} s^{-1}, 6 cool white fluorescent bulbs at 4100 K and 3 warm white fluorescent bulbs at 3000 K per incubator). To induce lipid production, cells were cultured to mid-log phase in regular TAP prior to deprivation of N by transfer to ammonium-free (i.e. nitrogen-free) TAP medium, as described previously [4]. Briefly, cells subjected to nitrogen deprivation were grown to 4×10^6 cells mL^{-1} and collected by centrifugation at 1006 xg for 5 min at room temperature. The supernatant was discarded, and the cells were washed in nitrogen-free TAP. Cells were then resuspended in nitrogen-free TAP to a final cell count of 2×10^6 cells mL^{-1}. Cell densities were determined using a hemocytometer.

[1] Knight, J. B., Vishwanath, A., Brody, J. P. & Austin, R. H. Hydrodynamic focusing on a silicon chip: mixing nanoliters in microseconds. *Physical Review Letters* **80**, 3863 (1998).
[2] Lee, G.-B., Chang, C.-C., Huang, S.-B. & Yang, R.-J. The hydrodynamic focusing effect inside rectangular microchannels. *Journal of Micromechanics and Microengineering* **16**, 1024 (2006).
[3] Laudon, M. Chlamydomonas resource center, university of minnesota. `http://chlamycollection.org/` (Data of access:20/07/2015). Online.
[4] Blaby, I. K. *et al.* Systems-level analysis of nitrogen starvation–induced modifications of carbon metabolism in a chlamydomonas reinhardtii starchless mutant. *The Plant Cell Online* **25**, 4305–4323 (2013).
